# Can *Urtica dioica* supplementation attenuate mercury intoxication in Wistar rats?

**DOI:** 10.14202/vetworld.2015.1458-1465

**Published:** 2015-12

**Authors:** Wafa Siouda, Cherif Abdennour

**Affiliations:** Department of Biology, Faculty of Sciences, Laboratory of Animal Ecophysiology, University Badji Mokhtar-Annaba, Annaba 3000, Algeria

**Keywords:** biochemical markers, fertility, glutathione, mercury, *Urtica dioica*, Wistar rats

## Abstract

**Aim:**

The objective of this study was to investigate the possible protective role of nettle *Urtica dioica* (UD) against Hg-induced toxicity.

**Materials and Methods:**

A total of 28 rats were equally divided into four groups: the control, the Hg (0.8 g HgCl_2_/kg in the diet), the UD (1.5 ml UD/rat by gavage), and the Hg+UD group. HgCl_2_ was daily dissolved in distilled water and immediately mixed with the standard diet. A solution of daily infused fresh nettle leaves in boiling water (16 g in 25 ml) was obtained and then it was administrated by gavage. Biochemical and reproductive markers, in addition to glutathione (GSH) level (liver, kidney and testis) and the histological profiles (testis and epididymis) were evaluated after 1 month exposure.

**Results:**

Compared to the control, the levels of glucose, triglycerides, urea, creatinine, alanine aminotransferase (ALT), aspartate aminotransferase (AST), and alkaline phosphatase (ALP) were significantly raised in the Hg group. In the latter group, the concentrations of Mg, Fe, and Ca were significantly decreased. Besides, Hg+UD group has only showed raised AST activity and reduced Mg level. Concerning the fertility markers, Hg has provoked a significant decrease in the spermatozoa’s concentration and motility and in plasma testosterone level as well. Furthermore, hepatic, renal and testicular GSH concentrations have declined significantly in the Hg treated rat compared to the control. A remarkable enhancement of the GSH level was observed in all organs of the UD group. The histological examinations of the Hg group have revealed marked testicular degeneration of the most seminiferous tubules, and showed few sperms in the lumen of epididymis ducts. However, the Hg+UD rats have demonstrated an improved histological structure with the presence of important numbers of sperms in the lumen. In addition, a clear stabilization of organized seminiferous tubules and an increased sperms’ numbers were noted in the UD supplemented rats.

**Conclusion:**

Nettle leaves have not only played a clear protective role during Hg intoxication, but it also enhanced hepatic, renal and testicular GSH level of Wistar rats.

## Introduction

Mercury has been regarded as a priority pollutant by many international agencies [1], because it is widely used in different fields of human life. It has been known that mercury toxicity could provoke neurological, digestive, hematological, renal, respiratory, immune, and reproductive disorders, which are dependent on the dose, the chemical form and the exposure route [2-4]. In fact, mercury has a high affinity and a stable complex to sulfhydryl groups and other biomolecules which might disturb some structures as enzymes [5] and metabolic processes [6]. Consequently, oxidative stress was proposed as one of the most mechanisms of Hg pathological exposure [7].

Healing plants’ extracts and their bioactive metabolites play important role in the cases of oxidative injuries, not only in the prevention of diseases, but also to treat them with proven efficacy. The stinging nettle *Urtica dioica* (UD) has a long history of therapeutic utilizations in folk medicine [8]. It is an annual herb that is widely distributed around the world [9], especially in the Mediterranean region. UD contains different beneficial compounds as minerals (iron, manganese, potassium, and calcium), vitamins (A, D and C), proteins, anti-oxidants, chlorophyll, carotenoids [10], flavonoids, fatty acids, and polysaccharides [11]. The aqueous and alcoholic extracts have been used for long time for the treatment of anemia [12], and applied as diuretic in the treatment of urinary, bladder and kidney dysfunctions [13]. Furthermore, its beneficial effects have been reported on inflammation, hypoglycemia, hypotension, benign prostatic hyperplasia [14], and liver failure [15] and acts as an antioxidant [16,17].

The objective of this study is to evaluate the beneficial use of UD, a local natural herb widely distributed, against chronic mercury intoxication of male Wistar rats, where some biochemical and fertility markers were investigated.

## Materials and Methods

### Ethical approval

The project of the PhD research program has been permitted by the Ethical Committee of Animal Sciences at the University of Badji Mokhtar-Annaba before starting the experimental work.

### Animals

Male adult Wistar rats weighting 165±10 g were obtained from Pasteur Institute, Algiers (Algeria). Animals were maintained in the Animal House of the Biology Department under controlled conditions, in which they subjected to the same conditions of light, humidity and temperature. Standard diet was supplied by “ONAB, rodent feed, Bejaia, Algeria” while food and water were provided *ad libitum*.

### Experimental design

About 28 rats were divided into four equal groups: the control, the Hg (0.8 g Hg/kg diet), the UD (1.5 ml UD/rat by gavage), and the Hg+UD group (0.8 g Hg/kg diet+1.5 ml UD/rat). Daily inorganic mercury (HgCl_2_) was dissolved in distilled water and then it was mixed with known quantity of diet.

The nettle UD was collected from clean area of Souk Ahras region (North-East Algeria) at the beginning of spring, and then it was identified by the department staff. Daily fresh leaves were infused in boiling water (16 g in 25 ml of distilled water) during 5 min to get a hot green solution, which was immediately filtered and left for about 15 min to be cooled, and then it was administrated to rats by gavage. Both mercury and UD solution were given at nearly 09:00 am for 7 days a week during a period of 30 consecutive days.

### Blood sampling

Blood was collected from anaesthetized rats by puncture of the jugular vein. Blood samples were immediately collected in labeled polypropylene test tubes containing heparin for biochemical studies. Blood was then centrifuged at 4000 rpm/min for 15 min, and then the plasma biochemical markers were evaluated.

### Weight assessment

Total body weight of each rat was measured weekly in the early morning over the experimental period of 1-month while food and water were measured daily at the same time.

### Biochemical analysis

Plasma biochemical markers were measured by an automated apparatus (Diatron PICTUS 200) where commercial “Spinreact kits, Spain” were used. Plasma testosterone concentration has been estimated by electrochemiluminescence immunoassay method using the automated apparatus (Cobas e 411).

### Fertility markers

The spermogram were realized according to the method of the World Health Organization [18] by making a small incision at the epididymis level to obtain semen. One drop of sperm of nearly 1 µl was added to 49 µl of physiological solution (0.9% NaCl), and then spermatozoa’s concentration and motility were estimated.

### Histological examination

After sacrifice, testis and epididymis were immediately collected and preserved in 10% neutral buffered formalin, where it was examined according to the classical method of Martoja and Martoja [19].

### Dosage of glutathione (GSH)

After removing organs (liver, kidney and testes), about 1 g of each one was homogenized in 2 ml of phosphate buffered saline. Homogenates were centrifuged at 10.000 g for 15 min at 4°C, and the resultant supernatant was used for the determination of reduced GSH [20], modified by Jollow *et al*. [21]. Total GSH content was expressed as nanomoles of GSH a milligram of proteins.

### Proteins estimation

Proteins were measured by the method of Bradford [22] using bovine serum albumin as a standard.

### Statistical analysis

The statistical analysis was achieved using one-way analysis of variance to compare between all groups, followed by Student’s t-test. The test was considered significant at p<0.05 level.

## Results

### Body weight

**Figure-1:**
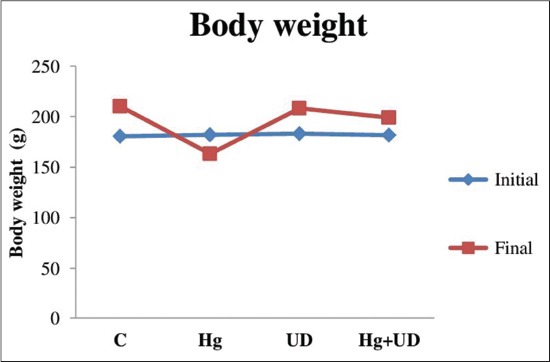
Initial and final total body weight (g) of males rats subjected to mercury and *Urtica dioica* during 30 consecutive days.

Final mean total body weights (182±2±23.5 g) after 30 days Hg exposure has decreased considerably compared to the initial weight (163.2±15.3 g) with a percentage of (−10.44%). Whereas, those of UD and Hg+UD mean total body weight have increased by (+13.66%) and (+9%), respectively. However, the final total body weight of the control has risen by (+16%) after 30 days. The data were presented in Figure-1.

### Clinical observations

During the period of mercury administration some adverse neurological and behavioral changes were observed. Rats have lost appetite relatively and they were anxious at the beginning of the experiment, then they started to be quiet thereafter due to tiredness and muscle weakness, while the respiration rate has risen. Rats’ unsteady walking is probably an indication of poor coordination. There was also a decrease in total body weight accompanied with a loss of skin hair in various body regions. Moreover, large fluctuation of food and water consumption was noticed, especially in the first few days. Contrary, the behavioral activities of the UD and the Hg+UD groups were comparable to the control.

### Biochemical markers

Data are presented in Table-1. The mercury-treated rats caused a significant elevation in the level of glucose, triglycerides, urea, creatinine, aspartate aminotransferase (AST), alanine aminotransferase (ALT), and alkaline phosphatase (ALP) when compared to the control rats. Contrary, results showed a significant decrease in the level of Mg, Fe, and Ca of the Hg treated group. In the combined treatment (Hg+UD), only AST and Mg levels were significantly different than that of the control. Creatinine, Mg and Ca concentrations were significantly different between all groups.

**Table-1:** Effect of UD on biochemical markers of rats after 30 days Hg intoxication.

Groups	Control	Hg	UD	Hg+UD
Glucose (g/L)	0.88±0.16	1.57±0.64*	0.80±0.10	0.86±0.16
Triglycerides (g/L)	0.32±0.02	0.59±0.10*	0.32±0.02	0.33±0.04
Urea (g/L)	0.25±0.06	0.47±0.06*	0.27±0.06	0.23±0.05
Creatinine (mg/g)	0.44±0.45^#^	0.81±2.64*^#^	0.40±0.73^#^	0.40±0.89^#^
AST (UI/L)	64.3±16.2	169.5±36.9*	66.9±15.2	105.0±16.5*
ALT (UI/L)	26.05±4.38	67.31±9*	25.95±3.18	26.86±6.03
ALP (UI/L)	118.9±20.6	229.0±20.8*	102.4±10.3	120.0±15.4
Mg (mg/dl)	3.30±0.63^#^	1.08±0.10*^#^	3.65±0.54^#^	2.49±0.53*^#^
Fe (µg/dl)	107.6±10.2	56.5±05.6*	109.5±13.7	102.9±11.1
Ca (mg/L)	82.60±6.07^#^	69.14±9.88*^#^	89.42±5.63^#^	76.20±12.40^#^

* Significantly different when compared to the control,

# Significantly different between groups. AST=Aspartate aminotransferase, ALT=Alanine aminotransferase, ALP=Alkaline phosphatase, UD=*Urtica dioica*

### Fertility markers

**Figure-2:**
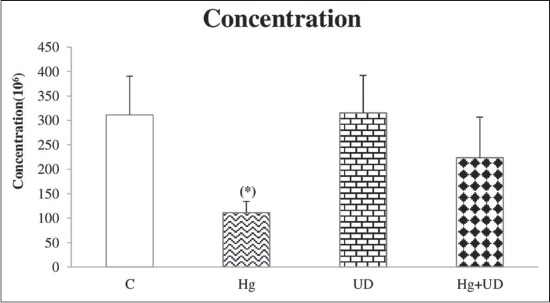
Spermatozoa’s concentration (10^6^) of males rats subjected to mercury and *Urtica dioica* during 30 consecutive days. * Significantly different when compared to the control.

**Figure-3:**
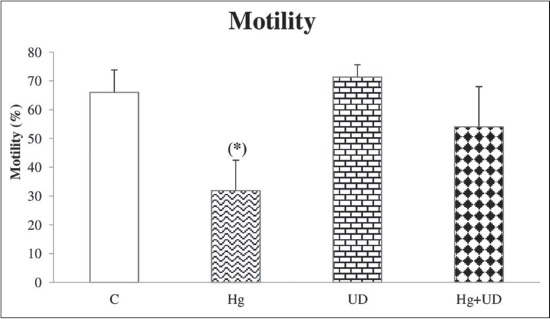
Spermatozoa’s motility (%) of males rats subjected to mercury and *Urtica dioica* during 30 consecutive days. *Significantly different when compared to the control.

**Figure-4:**
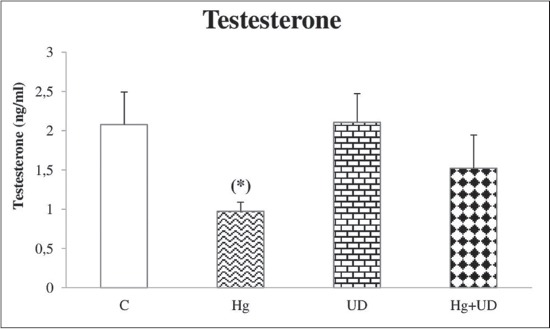
The concentration of testosterone (ng/ml) of male rats subjected to mercury and *Urtica dioica* during 30 consecutive days. *Significantly different when compared to the control.

Mean sperm concentration and motility were decreased significantly in rats exposed to mercury alone compared to the control. However, the concentration and motility of sperm cells were remarkably increased in both (UD) and (UD+Hg) groups in comparison with the control (Figures-2 and 3). Compared to the control, testosterone concentration has declined clearly in the Hg exposed group, but its decline was not statically significant in the combined treatment (Figure-4). The levels of fertility markers in the UD group were comparable to that of the control.

### GSH

**Figure-5:**
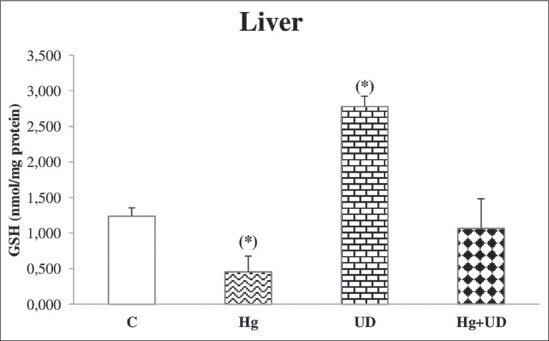
The level of hepatic glutathione (nmol/mg protein) of male rats subjected to mercury and *Urtica dioica* during 30 consecutive days. *Significantly different when compared to the control.

**Figure-6:**
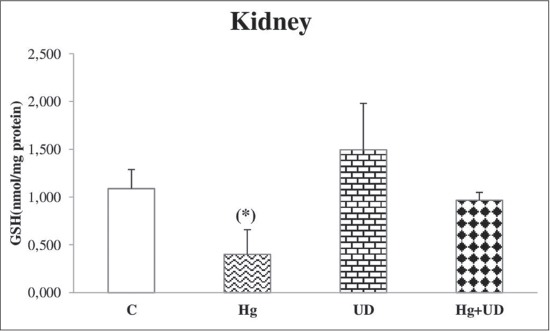
The level of renal glutathione (nmol/mg protein) of male rats subjected to mercury and *Urtica dioica* during 30 consecutive days. *Significantly different when compared to the control.

**Figure-7:**
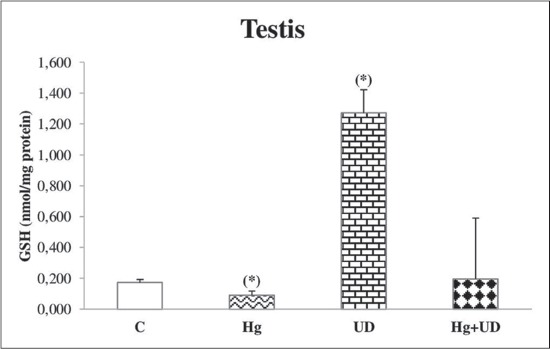
The level of testicular glutathione (nmol/mg protein) of male rats subjected to mercury and *Urtica dioica* during 30 consecutive days. *Significantly different when compared to the control.

A significant decrease of hepatic GSH level was observed in rats exposed to mercury, but GSH concentration was remarkably raised in the UD group, followed by Hg+UD group with less extent (Figure-5). Renal GSH concentration was significantly lower in the Hg exposed animals compared to the control and the UD group as well (Figure-6). Accordingly, there was a significant reduction in testicular GSH level of the Hg group compared to the control, while its level was remarkably higher in the UD treated rats (Figure-7). A large fluctuation of testicular GSH concentration between individuals of the Hg+UD group was seen.

### Histological profile

**Figure-8:**
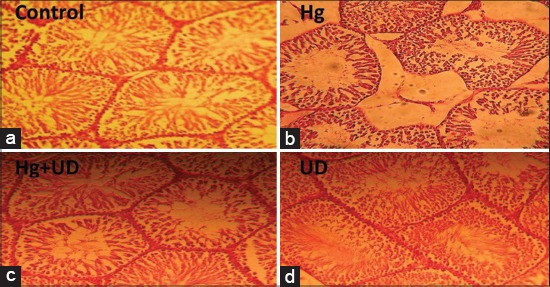
Histological profile of male rats testis showing the control showing normal histology, the *Urtica dioica* (UD), the Hg and the Hg+UD groups after 30 days treatment (×250), (a) Control: It has normal histological structure of active mature functioning seminiferous tubules associated with complete spermatogenic series, (b) Hg: It showed marked degeneration of most seminiferous tubules with absence of spermatogenic series in tubular lumen and a thickening of basal membrane, (c) Hg+UD: It showed normal histological structure of most seminiferous tubules, (d) UD: It showed and improved concentration of sperms and a clear stabilization of organized seminiferous tubules with many newly formed spermatogenic cells arranged properly in the tubules.

**Figure-9:**
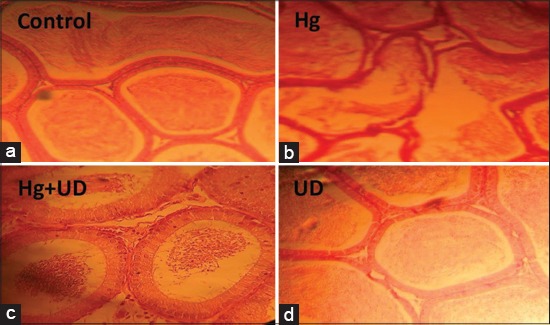
Histological profile of male rats epididymis showing the control, the Hg, Hg-*Urtica dioica* (UD) and UD the groups after 30 days treatment (×250), (a) Control: It showed normal histological structure, (b) Hg: It revealed few sperms in the lumen of epididymis ducts with irregular basement membrane disorganization and degeneration of some spermatogenic cells, (c) Hg+UD: It demonstrated nearly normal epididymis stucture with the presence of important numbers of sperms in the lumen, (d) UD: It showed an improved epididymis stucture with large numbers of sperms in the centre.

Figures-8 and 9 illustrated the histological examination of testis and epididymis tissues of different treatment groups. Microscopic assessment revealed the normal structure of seminiferous tubules, sperms with normal morphology and concentration in the control. The Hg has caused intoxication demonstrated by few numbers of spermatozoa in the tubules, different degrees of degeneration in the lining spermatogenic tubules, and delocalization of seminiferous tubules.

On the other hand, the toxic effects of mercury were reduced in testis of Hg+UD supplemented animals. In these circumstances, sperm with normal morphology, concentration and motility were observed and was close to the control group. Interestingly, rats supplied with a combination of Hg+UD or UD have a homogeneous and normal testis and epididymis tissue’s structures. The UD treated group showed stabilization of organized seminiferous tubules and many newly formed spermatogenic cells arranged properly inside the tubules better than the control group. Results obtained from the histological architecture were inconsistency with the reproductive markers as well as the GSH status.

## Discussion

The administration of Hg to rats showed a significant decrease of total body weight compared to the control. Such results might be related to the decrease of daily food and water consumption; which is in accordance with the report of National Toxicology Program Working Group [23]. Besides, in these experimental conditions, the presence of UD with Hg has caused an enhancement of rat body weight. Previously, UD antioxidants have been reported to eliminate free radicals produced [24] with no secondary effects [25].

In this study, the exposure of rats to Hg during 30 days has increased plasma glucose, but no difference was recorded in rats treated with Hg+UD. Previously, Hg intoxication was suggested to increase the energy supply to cope with the metal stress [26]. Interestingly, a remarkable hypoglycemia was registered in HgCl_2_ intoxicated rats, which was more obvious after 60 days exposure [27]. In this case, it seems that the treatment of diabetes with UD has reduced blood sugar to its normal level [28,29], and had hypoglycemic effect as well as through improving insulin secretion of hyperglycemic rats [30].

The actual results show that the concentration of triglycerides raised remarkably in the Hg treated group, which is not the case for the combined treatment of Hg+UD. The UD leaves contain chlorophyll, which has been proved to have hypolipidemic effects [31]. The UD extract has shown an apparent effect on animal models at doses of 100 and 300 mg/kg by reducing the levels of total cholesterol and low density lipoprotein, accompanied with a remarkable decrease of liver enzymes and total body weight when fed a high cholesterol diet [32].

This study demonstrates that the treatment of rats with Hg has led to a pronounced elevation of urea and creatinine concentration. It is well known that Hg administration could accumulate in renal tissues [33], and elevate these markers [34-36]. On the other hand, the supplementation of rats with Hg+UD or UD alone showed no noticeable change of urea and creatinine. Therefore, flavonoids and the high potassium content of UD may contribute to the diuretic action, which allows the body to excrete wastes including mercury, and that why stinging nettle is being used as a diuretic agent [14].

Results indicated a significant increase in plasma AST and ALT activities by mercury, but UD has preserved normal level of AST activity only. This increase is certainly come from damaging the plasma membrane permeability through the fixing of Hg ions to its proteins [37]. The increased in liver enzymes during Hg intoxication was already supported by Sheikh *et al*. [38] and Ejebe *et al*. [39]. After 5 weeks exposure to mercury chloride, serum AST activity was higher in rabbits compared to the group supplemented with a combination of *Pistacia lentiscus* oil and Hg [40]. The treatment with UD effectively protected rats against Aflatoxin-induced hepatotoxicity, as evidenced by the decreased AST and ALT activities [41]. Furthermore, UD treatment for 60 days has exhibited remarkable reduction in the liver enzyme levels and also has increased the antioxidant enzyme activities in tetrachloromethane-treated rats [42]. The present results are in accordance with the reported results concerning the protective role of UD [43].

The results of this work indicated the clear effect of Hg on plasma ALP activity. This enzyme is liberated into the blood when parenchymal liver cells are damaged. It is reported that the treatment of rats by HgCl_2_ for 6 months had increased the activity of ALP [23]. In addition, Rao and Sharma [44] have observed a reduction in mice ALP activity treated with HgCl_2_ for 45 days. Contrary, no significant change was reported in the group fed with Hg and UD. Accordingly, the efficiency of blue-green algae *Spirulina fusiformis* was obvious to protect ALP activity from Hg toxicity in mice [45].

The current results showed a significant decrease in the levels of Mg, Fe and Ca of rats exposed to Hg. Meanwhile no variation was noted concerning these minerals in the group treated with Hg+UD. This lowering effect was happened probably because Hg is known to cause lack of appetite [46], as well as it is able to provoke many dysfunctions in the absorption of nutrients [47]. However, no changes in the levels of Mg, Fe and Ca was seen in rats supplemented with UD alone, possibly that is due to the high content of minerals and vitamins found in different parts of this herb [9], especially in the fresh leaves [48].

Mercury exposed rats of the present study indicated a reduction in the concentration and motility of epididymal sperm. Such results are in agreement with that of Pb on rabbit reproductive system [49]. Accordingly, another study revealed that epididymis sperm count and motility were markedly reduced by HgCl_2_ [50]. The latter has led to spermatozoa’s damage, human infertility [51] and affected mice reproductive performance [52]. Thus, the epididymis is known to play an important role in providing the microenvironment for sperm maturation and storage. Consequently, the decrease in epididymal number/motility of sperm could be explained on the basis that Hg has crossed the blood-testis barrier and gained access to germinal cells, leading to testicular dysfunction [53]. Mammalian spermatozoa are rich in polyunsaturated fatty acids which are very vulnerable to reactive oxygen species (ROS) attack; the latter induce a decrease of sperm motility, presumably by a rapid loss of intracellular adenosine triphosphate, causing testicular damage [54]. Meanwhile, noticeable improvements of all sperm’s markers were recorded when UD was supplemented alone or combined with Hg, which certainly suggests the protective roles played by this wild herb. Apparently, UD contains many minerals and vitamins, especially vitamin C and E, as well as flavonoids [11], which can act as antioxidants to counteract Hg toxicity. Furthermore, many studies revealed that animals receiving vitamin E alone or combined with HgCl_2_ had sperm markers similar to the control values [49].

In this study, Hg has reduced testosterone concentration deeply after 1 month exposure. Hg intoxication may have affected testosterone synthesis at the level of Leydig cells. The supplementation of rats with UD alone or combined with Hg showed noticeable increase in testosterone level, which is in line with the results of mice exposed to HgCl_2_ and vitamin E together [44]. The variation in testosterone level of this study totally agree with the result of El-Boushy *et al*. [55] and Nagwa *et al*. [56] who observed a reduction in plasma testosterone concentration under Hg exposure, but it was different than that of workers chronically exposed to mercury vapor [57].

The testicular and epididymal histoarchitecture of Hg-treated animals from this study showed marked alterations characterized by the presence of different degrees of degeneration in the lining of spermatogenic tubules. Such lesions are attributed to Hg effect on endothelial cells, leading to its damage and histological deformation [55]. These overall changes of the histological profiles were attributed to ROS formation leading to the impairment of several cell membrane components. The degenerative status observed is in support with the biochemical data and oxidative stress markers. Interestingly, rats supplied with a combination of Hg+UD or UD alone have a homogeneous and normal testicular and epididymal tissue structure. Since UD leaves contain various minerals as iron and vitamins A, which have been identified for regulating the differentiation of epithelial cells, it seems that the sperm cells in group receiving the extract of UD were rapidly differentiated and released from tubules [58], leading to increased diameters of seminiferous tubules.

In the present investigation, the GSH level showed a noticeable significant hepatic, renal and testicular depletion the following Hg exposure. These results were parallel to Sheikh *et al*. [38] and Syversen and Kaur [59] who reported that exposure to HgCl_2_ were significantly decreased the reduced GSH and various antioxidants. Such depletions are likely related to the high affinity of Hg ions to thiol groups, as a result Hg could disturb cell functions [60]. It has been recommended that antioxidants could be valuable to the treatment of Hg injuries [61], therefore, the metal-GSH conjugation manner is necessary in the elimination of Hg into the bile. Hg was reported to cause remarkable exhaustion of GSH in the liver [62], testis [63] and other cell types [64]. Moreover, GSH-Hg complexes have been found in liver, kidney, and brain, and seemed to be the primary form in which Hg is transported and eliminated from the body [65]. On other hand, the co-administration of UD with Hg has resulted in keeping GSH at the control level. GSH is the main thiol antioxidant and the conjugating agent; it was known to bind electrophilic molecular species and free radicals intermediate. The supplementation of UD extracts demonstrated an antioxidant and hepatoprotective effect against anxious stimulus by tetrachloromethane [56]. Many studies proved that the flavonoids and phenols are good antioxidant against Hg-induced patho-toxicity and also act as effective chelators for several toxic metal ions [66]. Antioxidants present in UD infusion might have a defensive antioxidant and metal-chelating properties against Hg tissue injuries [43].

## Conclusion

From this investigation, Hg has altered many biological markers of rats including GSH. The histological structure of liver, kidney and testes were also affected. However, the consumption of UD along with Hg has helped preventing such markers, especially spermatozoa’s concentration and motility. Therefore, fresh nettle leaves are cheap natural protective herb that may play a beneficial role in the prevention of Hg intoxication.

## Authors’ Contributions

WS has carried out the experimental work in the AE laboratory and then she prepared the manuscript, while CA has checked the manuscript and polished English language. Both authors read and approved the final manuscript.
